# A multi-year analysis of acoustic occurrence and habitat use of blue and fin whales in eastern and central Fram Strait

**DOI:** 10.1371/journal.pone.0314369

**Published:** 2024-11-26

**Authors:** Marlene Meister, Elena Schall, Robert Dziak, Stefanie Spiesecke, Karolin Thomisch

**Affiliations:** 1 Ocean Acoustics Group, Alfred Wegener Institute Helmholtz Centre for Polar and Marine Research, Bremerhaven, Germany; 2 NOAA/Pacific Marine Environmental Laboratory, Newport, Oregon, United States of America; University of Maryland Center for Environmental Science, UNITED STATES OF AMERICA

## Abstract

Climate change-induced habitat alterations in the Arctic Ocean are expected to affect spatial and temporal occurrence patterns of seasonally migrating baleen whale species, leading to poleward range shifts and prolonged stays in Arctic waters. The aim of this study was to investigate occurrence patterns of blue (*Balaenoptera musculus musculus*) and fin (*B*. *physalus*) whales in Fram Strait, a summering habitat and historic whaling ground for both species. Passive acoustic monitoring data were collected between 2012 and 2021 at eight different mooring positions in eastern and central Fram Strait. These data were initially analyzed for the acoustic presence of blue and fin whales. Afterwards, the environmental impact on acoustic occurrences and the potential temporal expansion of acoustic presence periods were investigated. Blue whale acoustic presence showed a clear seasonality, with most calls being detected from July to October. Additionally, sporadic blue whale calls were detected in winter in three years on one or a few consecutive days. Fin whale acoustic presence varied between years, with peak presence from mid-summer through autumn, moderate presence in winter, and sporadic presence in spring. Random forest models suggested that zooplankton mass content, sea surface temperature and day of the year influenced blue whale acoustic presence, while fin whale acoustic presence was impacted by day of the year. For both species, no temporal trend in the onset and offset of acoustic presence periods was found. Our findings highlight eastern Fram Strait as key marine mammal habitat, probably serving as a feeding ground. Occasional (blue whales) and regular (fin whales) acoustic presence during the winter months further supports the hypothesis of complex migration behaviors in both species.

## Introduction

The Arctic Ocean is undergoing rapid and profound shifts due to climate change, with severe consequences for its marine mammal populations [1–6]. Rising water temperatures, sea-ice decline, and food web alterations directly and indirectly impact the habitat suitability for Arctic endemic and seasonally migrating marine mammal species [7–10]. Furthermore, with cross-Arctic shipping routes becoming more accessible as sea-ice declines, human activities in the Arctic Ocean are increasing [11, 12]. This puts marine mammals at a greater risk of noise pollution, habitat disruption, ship strikes, and oil spills [13, 14].

Habitat changes in the Arctic Ocean are expected to significantly affect the occurrence and abundance of seasonally migrating baleen whale species [7, 10, 15]. Diminished sea-ice cover and increased prey availability might affect their spatio-temporal distribution patterns, causing poleward range shifts and extended stays at Arctic feeding grounds [16, 17]. This might also result in prolonged temporal overlap and increased interspecific competition between Arctic endemic and seasonally migrating baleen whale species [7, 10, 17]. Furthermore, expanding presence of killer whales in the Arctic Ocean due to sea-ice decline may increase predation pressure on marine mammal species [18].

Blue (*Balaenoptera musculus musculus*) and fin whales (*B*. *physalus*) seasonally migrate into the Atlantic part of the Arctic Ocean [19–21]. Both species were heavily targeted during the era of commercial whaling, leading to a significant reduction in their population sizes [22]. Abundance estimates for the Central North Atlantic suggest blue whales still have a relatively low population size (∼3,000 individuals), while fin whales show signs of recovery (∼37,000 individuals) [23].

Historic whaling records provide insight into blue and fin whale migration from the North Atlantic into higher latitudes [22]. Blue whale migration is traditionally depicted to be very regular, involving a northward journey to waters north of Iceland for feeding in spring and a southward return to unknown breeding grounds in autumn [24, 25]. In contrast, fin whale migration is described to contain a certain amount of north-south movement, but with movements in general being more irregular compared to other baleen whale species [25–27]. Notably, fin whales were observed in Norwegian and adjacent waters throughout most of the year and were caught off the southwest Norwegian coast during winter [27]. Furthermore, part of the fin whale population was hypothesized to spend the winter in the Arctic Barents Sea [24], however, this idea was doubted by some whalers [27].

Fram Strait, situated between Greenland and Svalbard, is seasonally frequented by both blue and fin whales [19–21, 28]. The water passage features a distinctive current system, where the West Spitsbergen Current brings Atlantic waters northward, and the East Greenland Current carries Arctic waters southward, fostering large zooplankton abundances [29, 30]. High prey availability is likely to attract baleen whales [31], including blue and fin whales. Studies on seasonal occurrence of blue whales in western and central Fram Strait indicate their primary presence from August to October [19, 32]. Fin whales are suggested to mainly inhabit the region from July to December [19], with evidence of a part of the population residing year-round in western Fram Strait and in Svalbard Archipelago fjords [19, 33]. However, information on seasonal occurrences of blue and fin whales in eastern Fram Strait remains limited [28, 34].

Occurrence patterns of blue and fin whales in Fram Strait might already be influenced by climate change [19, 33] and are likely to undergo significant alterations in the future. In recent decades, the upper water layers of both the West Spitsbergen Current and the East Greenland Current have undergone significant warming [35, 36]. Simultaneously, distributional shifts among seasonally migrating baleen whale species, such as blue, fin, humpback (*Megaptera novaeangliae*) and minke whales (*B*. *acutorostrata*), were observed in the Svalbard Archipelago, with animals transitioning from the continental shelf break towards coastal areas [20]. Furthermore, interannual increases in call rates at the beginning of seasonal acoustic presence periods of blue whales in western Fram Strait suggest an extended temporal presence in recent years [19]. To effectively monitor climate-change induced variations in the occurrence of blue and fin whales, it is essential to conduct long-term studies that establish baseline information and uncover ongoing changes. To this end, passive acoustic monitoring (PAM) offers a non-invasive tool for continuous monitoring of vocalizing marine mammals. PAM overcomes challenges for visual studies especially in polar regions, such as harsh weather, limited daylight, and sea-ice coverage [e.g. 19, 37, 38]. Species-specific vocalizations, i.e. AB-calls produced by blue whales [39] and 20 and 130 Hz calls produced by fin whales [40, 41], allow for prompt and reliable species identification, making both species well-suited for investigation through PAM.

The objective of our study is to examine long-term acoustic occurrences of blue and fin whales in the Arctic Ocean using data derived from PAM in the shelf areas of eastern Fram Strait and in the deep waters of central Fram Strait. To this end, we analyze passive acoustic data collected between 2012 and 2021 and investigate the impact of environmental variables, including sea-surface temperature (SST), sea-ice concentration, and zooplankton mass content, on acoustic presence. We further explore potential temporal expansion of acoustic presence periods of both species. Our research aims to extend current knowledge on blue and fin whale seasonal occurrence in an important former whaling ground beyond previous studies in the western Fram Strait [19, 32] and Svalbard Archipelago fjords [33, 42]. Thereby, we seek to contribute to monitoring how global warming could affect distribution patterns of baleen whale species that seasonally migrate from the North Atlantic into the Arctic Ocean.

## Methods

### Data collection

PAM data were collected between 2012 and 2021 at eight distinct locations in eastern and central Fram Strait. Data were collected through the Ocean Observing System FRAM (Frontiers in Arctic Marine Monitoring [43]) by the Alfred Wegener Institute Helmholtz Centre for Polar and Marine Research (AWI), as well as by the National Oceanic and Atmospheric Administration Pacific Marine Environmental Laboratory (NOAA PMEL) via several research vessels, including RV Polarstern [44]. AWI devices consisted of SonoVault recorders (manufactured by develogic GmbH, Hamburg, Germany [37]) and AURAL recorders (Autonomous Underwater Recorder for Acoustic Listening, Model 2, Multi-Électronique [45]). NOAA PMEL devices consisted of autonomous underwater hydrophones (manufactured by Oregon State University and NOAA PMEL [28]).

**Fig 1:**
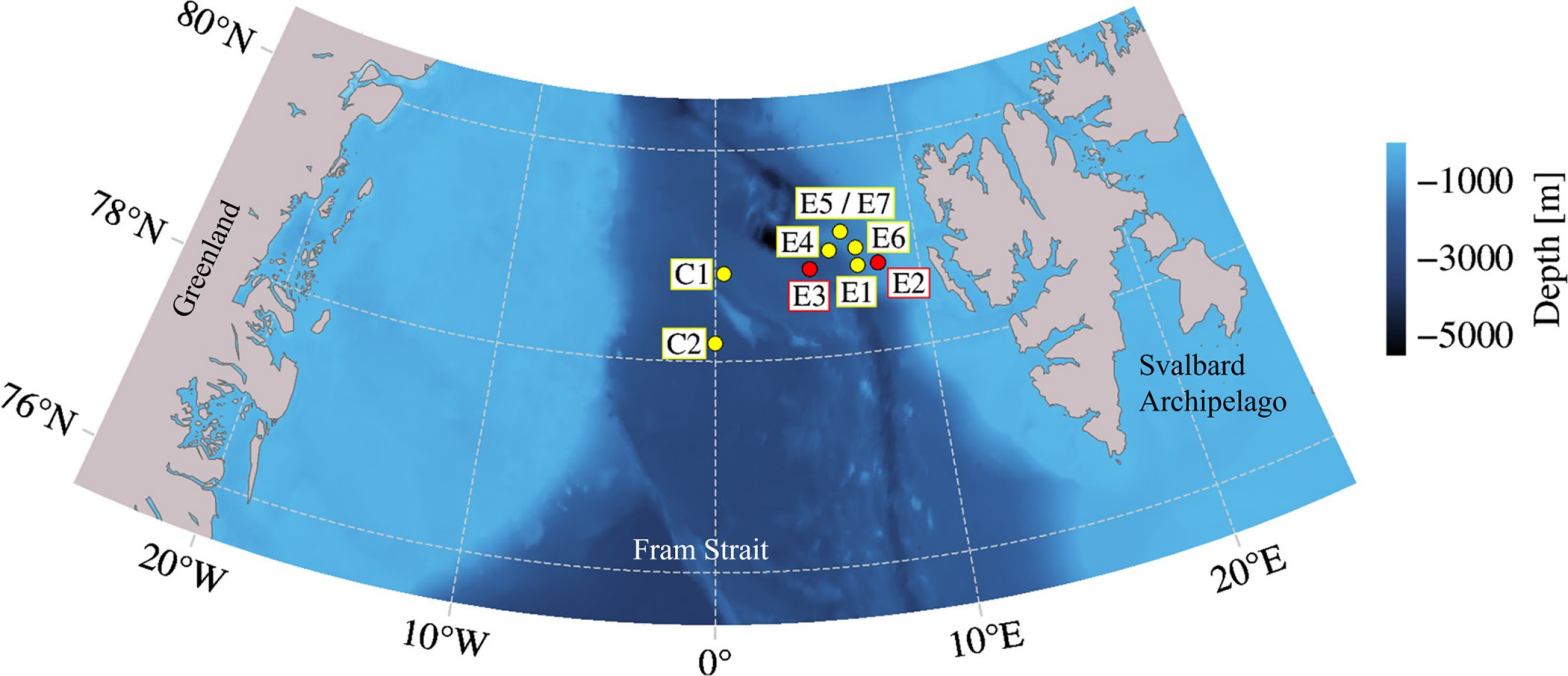
Recorder positions in eastern and central Fram Strait. Yellow dots indicate instruments owned by AWI, red dots indicate instruments owned by NOAA PMEL. Bathymetry map was created in PyGMT v0.9.0 [46] using the SRTM15+V2.6 grid [47].

In total, we selected nine recorders for this study, taking into account both data quality and the time period covered by the recordings (Fig 1, Table 1). Data selection involved visual examination of long-term spectrograms for all available FRAM recorders from AWI (S1 Table), accessible through the Open Portal to Underwater Soundscapes (OPUS, https://opus.aq/). We assigned an identification code to each recorder, including a letter indicating the relative site in the survey area (i.e. E for eastern Fram Strait and C for central Fram Strait) and a number indicating the order of recording start at each site. Seven of these recorders were owned by AWI (E1, E4, E5, E6, E7, C1, C2; further referred to as AWI data) and two were owned by NOAA PMEL (E2, E3; further referred to as NOAA PMEL data).

**Table 1:** Locations and recording settings of passive acoustic recorders deployed in Fram Strait between 2012 and 2022. ID is depicted using an identification code indicating location (E = eastern Fram Strait, C = central Fram Strait) and a number indicating the recording start order at each site.

Recorder ID	Mooring ID	Recorder Serial Number	Latitude [°]	Longitude [°]	Deployment period	Recording period	Water depth [m]	Recorder depth [m]	Sample rate [kHz]	Effective bandwidth [Hz]	Duty cycle	Data origin	Reference
E1	ARKF04-15	SV1026	78.83	7.00	06.2012–06.2015	06.2012–11.2012	1420	743	5.333	8–2666	continuous	AWI	[53]
E2	ARKF03-15	AUH033	78.83	8.00	06.2012–06.2015	07.2012–07.2014	1005	493	2	5–840	continuous	NOAA PMEL	[54]
E3	ARKF06-17	AUH012	78.84	4.65	10.2015–07.2016	10.2015–07.2016	2726	503	5	5–840	continuous	NOAA PMEL	[54]
E4	ARKF05-17	SV1088	79.00	5.67	07.2016–09.2018	07.2016–07. 2017	2100	808	48	8–24000	continuous	AWI	[55]
E5	ARKF04-OZA	SV1096	79.17	6.33	09.2018–07.2020	09.2018–08.2019	1418	836	48	8–24000	continuous	AWI	[56]
E6	ARKF04-19	SV1088	79.00	7.00	08.2019–06.2021	09.2019–12.2020	1218	805	48	8–24000	10min per 20 min	AWI	[57]
E7	ARKF04-OZA2	AU0302	79.17	6.33	07.2020–07.2022	07.2020–05.2021	1422	300	32	10–16000	10min per h, continuous	AWI	[58]
C1	ARKF16-09	SV1021	78.83	0.43	06.2012–09.2014	06.2012–11.2012	2525	800	5.333	8–2666	continuous	AWI	[59]
C2	ARKR01-01	SV1097	78.17	0.00	08.2016–07.2018	08.2016–08.2017	3013	752	48	8–24000	continuous	AWI	[60]

All devices were moored at depths around 500 to 800 meters, except for recorder E7, which was located at a depth of 300 meters (Table 1). Sampling rates between 2 kHz and 48 kHz were used for the recorders. AWI data were stored on internal memory cards at 10-minute intervals with a 24-bit resolution, and NOAA PMEL data were stored at 60-minute intervals with a 16-bit resolution. Seven of the recorders (E1, E2, E3, E4, E5, C1, C2) operated continuously. One recorder (E6) was set to a 50% duty cycle, sampling 10 minutes per 20 minutes. Another recorder (E7) was set to a duty cycle of 10 minutes per hour, however, due to an internal software issue, it switched to continuous recording in January 2021. Most recorders operated for at least nine months, except for E1 and C1 which stopped after five months due to battery depletion. One recorder (E1) also lacks 12 days of data in October due to a faulty memory card, and one recorder (E7) failed to record on the 29^th^ of February. All technical details are given in Table 1.

### Data preparation

Raw audio data were prepared and standardized following the data management workflow described in the AWI’s Standard Operating Procedures for passive acoustic data (SOPs, [48, 49]). Spectrograms of the audio data were created for display via OPUS and the data underwent quality control in line with AWI’s SOPs [50]. PAM datasets were published under license CC BY 4.0 according to FAIR principles via the data repository PANGAEA [51] and the NOAA National Centers for Environmental Information [52].

### Data analysis

A total of 3017 days of passive acoustic data were examined for the presence of Atlantic blue whale AB calls, fin whale 20 Hz pulses, and fin whale 130 Hz pulses. The data were re-sampled to a sampling rate of 500 Hz for data standardization to ensure uniformity and expedite processing. Atlantic blue whale AB calls and fin whale 20 Hz pulses were detected using two automatic methods followed by manual post-processing, while fin whale 130 Hz pulses were identified entirely through manual examination. Full descriptions of these methods are provided in the sections below. All manual data analysis was conducted in Raven Pro 1.6.4 (Bioacoustics Research Program, Cornell Lab of Ornithology, Ithaca, United States, https://www.ravensoundsoftware.com/) using consistent spectrogram settings for all analytical procedures (window size of 650 samples, Hann window, 50% overlap, DFT size of 1024 samples).

#### Test dataset

Before applying the automatic detection methods to the entire dataset, we compiled and manually analyzed a test dataset to (i) determine the input parameters for the fin whale detector and (ii) assess the performance of both the blue and fin whale detector. To compile this test dataset, we selected two days per month, analyzing every second file from each day. Whenever possible, we gave preference to the 1^st^ and 15^th^ day of each month, however, if these days were unavailable, we selected two other days randomly.

Please note that the test dataset was processed in two different ways due to data availability: (i) Determining input parameters for the fin whale detector: We only included days selected from AWI data and visually examined these files for fin whale 20 Hz pulses on a single-call basis. We did not include NOAA PMEL data here, as it became available at a later stage. (ii) Assessing detector performance: To evaluate the performance of both the blue and fin whale detectors, we selected days from both AWI and NOAA PMEL data. We analyzed these days at the file level (i.e., 10-minute intervals) for Atlantic blue whale AB-calls and fin whale 20 Hz pulses, with one call per file representing the acoustic presence of the respective species. This complete test dataset accounted for approximately 3.6% of the entire dataset.

#### Atlantic blue whale AB calls

The analysis of blue whale acoustic occurrence involved a two-step process, consisting of an initial application of a highly sensitive automatic detection method to the complete dataset, followed by a manual false positive control on all detections. The idea behind this approach was to minimize any potential bias that could come from relying solely on manual classification methods [61] and to expedite the process, rather than aiming to be a fully autonomous detection method. For automatic detection, we used the built-in ‘Band Limited Energy Detector’ tool of the Raven Pro software [62]. The detector was set up to identify A-, B-, and AB- calls, without differentiating between call types.

We determined input parameters describing the target sounds, including minimum frequency of 16 Hz, maximum frequency of 18 Hz, as well as minimum duration of 5.2 s and maximum duration of 20.15 s, similar to findings from existing literature [39, 63]. Additional input parameters, including minimum separation and parameters related to noise control (S2 Table), were set in accordance with the Raven software manual provided by Cornell Lab of Ornithology [64]. We applied no bandwidth filter or exclusion band to increase the sensitivity of the detection method. After processing the test dataset, to evaluate the detector’s performance, we compared the results of blue whale acoustic presence obtained through manual analysis with the results obtained through automatic detection per hour (S3 Table). The detector demonstrated robust sensitivity, surpassing 94% for the majority of recorders. Only E3 exhibited a lower sensitivity of 78%, primarily due to the limited occurrence of blue whale calls in the data from this recorder. After successful performance evaluation, we applied the detector to the entire dataset, and logged the files with no call detections as blue whale acoustic absence. The remaining files underwent manual false positive verification, where we checked each file, and retained those containing at least one blue whale vocalization as confirmed blue whale acoustic presence. Manual validation identified anthropogenic noise, such as ship noise and signals from seismic operations, as a source of false positive detections.

#### Fin whale 20 Hz pulses

To examine the presence of fin whale 20 Hz pulses, we applied an automatic detection algorithm developed by Schall and Parcerisas 2022 [65]. The detector processes audio data in 2-second segments (with 1.5 s overlap), applies bandpass filtering, and computes metrics including kurtosis, temporal and spectral signal-to-noise ratio, and bandwidth for presumed fin whale 20 Hz pulses within each segment. To avoid multi-detection of the same pulse, detections within a 2-second period are merged, keeping the earliest timestamp as well as the highest values for kurtosis, signal-to-noise ratios, and bandwidth. Users can then define specific threshold values for these metrics and utilize a minimum detection count per unit of time to filter detection results. In our study, the application of the detector comprised (i) an initial detection run on the test dataset, (ii) the evaluation for optimal threshold combinations and detector performance, (iii) applying the detector to the complete dataset, and (iv) manually controlling for false positives in detections.

We conducted the initial run of the detector in MATLAB R2023a on the test dataset. Within the detector settings, we calibrated the bandpass filter ranging from 18 to 28 Hz, with lower limit set at 18 Hz to minimize interference from Atlantic blue whale AB calls [39]. Additionally, for the spectral SNR calculation we defined the lower noise band from 11 Hz to 15 Hz and the upper noise band from 30 to 33 Hz.

To determine optimal threshold values, we compared computed metrics from the initial detection run with the manual annotations of single 20 Hz pulses in the test dataset. We used manual annotation timestamps with a ±1.5-second buffer and identified detector timestamps falling within these intervals. We then extracted the metrics (kurtosis, temporal and spectral signal-to-noise ratio, and bandwidth) for all detector timestamps. We generated histograms for all these extracted metrics for both the timestamps with and those without manual annotations (S1 Fig). Based on these histograms, we assessed the range of each metric and selected potential threshold values at regular intervals (S4 Table). We tested all possible combinations of thresholds (in total 21,870 combinations) by comparing respective detections with the ground truth. The optimal threshold combination for each recorder was set at a minimum true positive rate of 0.7 and the smallest false positive rate possible. The evaluation revealed fairly similar optimal threshold values for five recorders (E1, E5, E6, E7, C1 = group 1), whereas two recorders (E4, C2 = group 2) displayed significantly lower values for the spectral signal-to-noise ratio (i.e., due to an additional unknown interfering noise band in these data; S5 Table). Consequently, we established two distinct groups and determined an optimal threshold combination for each group, incorporating all respective recorders (S6 Table).

To evaluate the performance of the detector on the basis of hourly presence of 20 Hz pulses, we filtered computed metrics of the initial detection run per group with respective threshold combination and compared filtered metrics with manual annotations (S7 Table). For NOAA PMEL data, which were not included into previous optimal threshold determination, we used both combinations and compared detector performance on an hourly basis. Subsequently, we assigned each recorder one group (E2 = group 1; E3 = group 2), ensuring a true positive rate exceeding 0.7 while minimizing the occurrence of false positives (S7 Table).

Once we determined the optimal threshold combinations and assessed performance, we ran the detector on the entire dataset. Afterwards we filtered computed metrics with the respective threshold combination for each deployment according to the group they were assigned to during the test. Since performance evaluation did not yield satisfactory results in terms of false positives (precision < 0.63 for all recorders, S7 Table), we conducted a manual false positive control, in which we examined each file containing a detection for the presence of at least one 20 Hz pulse. Manual validation found underwater sound, like ship and seismic signals, to be a cause of false positives.

#### Fin whale 130 Hz pulse

Accompanying fin whale 20 Hz pulses, there is often a concurrent high-frequency component, notably a short upsweep centered around 130 Hz in Northern Atlantic fin whales [41, 66]. The presence of this 130 Hz pulse was estimated on hourly basis via manual analysis by a trained human analyst using Raven Pro 1.6.4. We decided to include all files into manual analysis, not just those with previously detected 20 Hz pulses, due to the prevalence of ship noise potentially masking 20 Hz pulses. For further analysis, we classified an hour as indicating fin whale acoustic presence if it contained at least one 20 Hz pulse (with or without an accompanying 130 Hz pulse), or at least one single 130 Hz pulse.

#### Environmental data

Daily SST with a resolution of 0.05° x 0.05° from satellite measurements was sourced from Copernicus Climate Change Service [67]. Daily sea-ice concentration with a resolution of 3.125 x 3.125 km from satellite measurements [68] was sourced from the University of Bremen (https://data.seaice.uni-bremen.de/amsr2/asi_daygrid_swath/n3125/). Daily zooplankton mass content (expressed as carbon in sea water) with a resolution of 0.083° × 0.083° from model predictions [69–71] was sourced from the Copernicus Marine Data Store [72].

We calculated daily mean values of each environmental variable by establishing species-specific detection radii around each mooring position and extracting data points falling within these radii. Maximum possible detection ranges for blue and fin whales in both shelf and deep-water areas in Fram Strait have been previously modeled by Ahonen et al. 2021 [19]. Based on these findings, we expected detection radii as follows: For recorders positioned in eastern Fram Strait, which primarily comprises shelf areas, we assumed detection radii at 60 km for blue whales and 30 km for fin whales. In contrast, for recorders situated in central Fram Strait, characterized by deep-water conditions, we assumed detection radii at 100 km for blue whales and 55 km for fin whales. To extract environmental information, we calculated geodesic distances between recorder locations and all data points and retained only points within the specified radii.

#### Habitat use and seasonal presence analysis

We investigated individual effects of different variables on the acoustic presence of blue and fin whales (measured in hours per day) using random forest models [73]. We chose the random forest model due to its wide usage and robust predictive capabilities, as well as its suitability for handling complex interactions and highly correlated predictor variables [74–76]. PAM data for model development comprised our complete dataset, with recordings from both eastern and central Fram Strait. We included three environmental variables in the models, i.e. SST, sea-ice concentration, and zooplankton mass content, which were reported to influence the acoustic occurrence of blue and fin whales [e.g. 77–80]. By also including day of the year (DOY), we further investigated potential seasonal patterns. Random forest models for both species were implemented in Python 3.11 using RandomForestRegressor from sklearn.ensemble. Dataset partitioning involved a 70% - 30% split into training and validation sets. Hyperparameter tuning encompassed defining parameter ranges (S8 Table) and executing a grid search with 5-fold cross-validation to identify optimal parameter combinations maximizing model performances. To mitigate overfitting risks, we restricted the maximum tree depth to five, and evaluated hyperparameters through cross-validation curve analysis. Model performance was assessed on the validation datasets kept separate from the model tuning process. We extracted feature importance scores from trained random forest models and further examined feature importance by substituting one or more variables with random values to explore impacts on model performances.

To explore potential temporal expansion of seasonal acoustic presence of vocalizing blue and fin whales, we combined all data from eastern Fram Strait into a single multi-year time series, due to the limited temporal coverage at individual mooring positions. Data from central Fram Strait were not included in this analysis. We then determined the ordinal date at which the seasonal vocal activity was first recorded (further referred to as onset) and the ordinal date at which seasonal vocal activity was last recorded (further referred to as offset). To this end, we calculated the cumulative distribution of days with acoustic presence for a defined time period per year and recorder following the approach by Hauser et al. 2017 [81, S2 Fig]. We defined onset as the 5% quantile and offset as the 95% quantile of the cumulative distribution of days with acoustic presence in each time period. Analyzed days included May 1^st^ to July 31^st^ for blue whale onset; October 1^st^ to November 30^th^ for blue whale offset; 1^st^ of June to 31^st^ of July for fin whale onset; and 1^st^ of January to 31^st^ of May for fin whale offset. We determined these time periods to align closely with expected event occurrences, reducing data gaps and thereby making the acoustic data more comparable across years. We calculated the onset and/or offset for all years and recorders from eastern Fram Strait where data were available one month before (for onset) or after (for offset) each time period. Final data selected for analysis exhibited no temporal overlap, facilitating their aggregation into a single multi-year series.

## Results

### Spatial and temporal patterns

**Fig 2:**
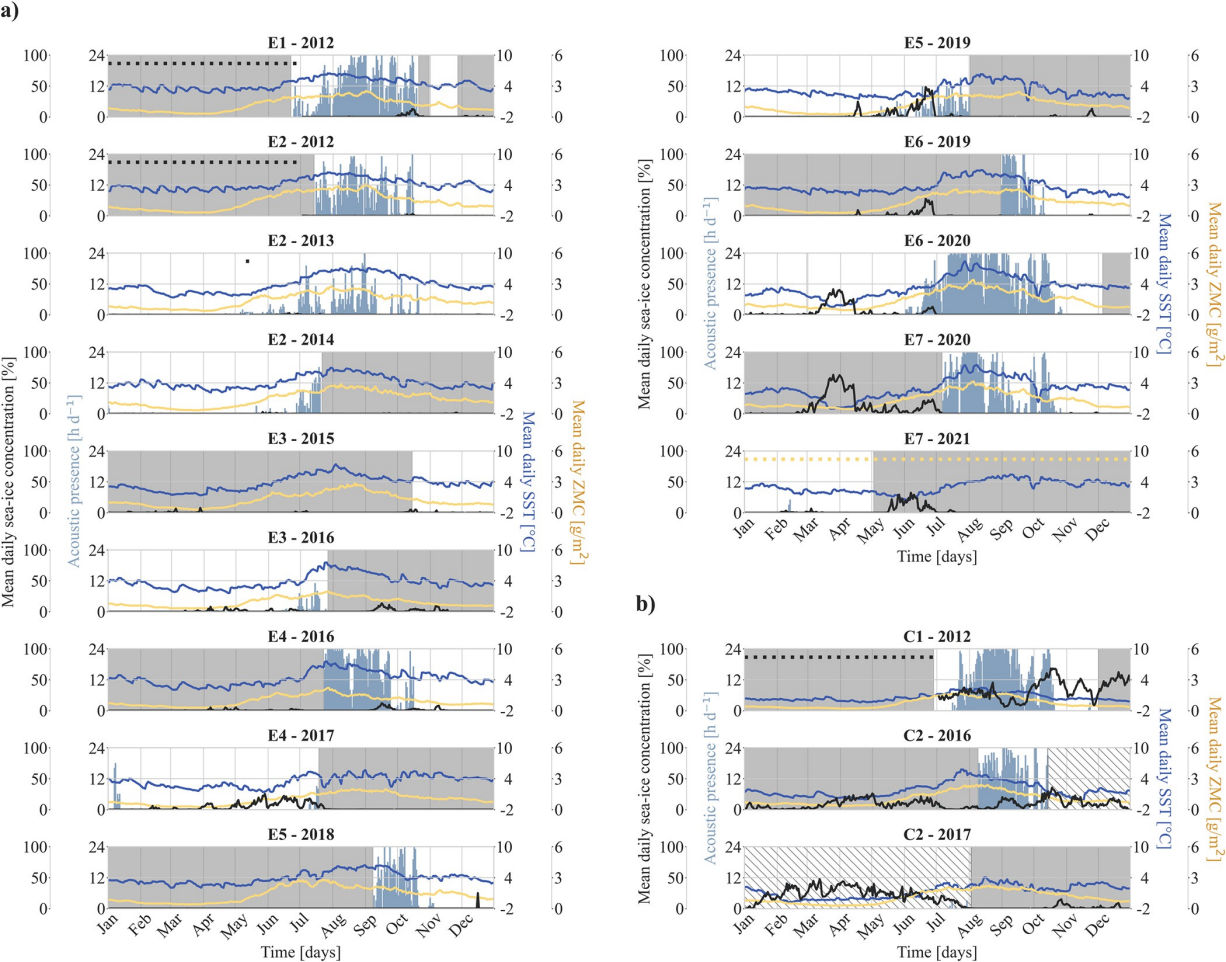
Interannual patterns in acoustic presence of blue whales (AB-calls) in a) eastern and b) central Fram Strait. Shaded periods denote missing acoustic data, while hatched areas indicate acoustic data that are presumably compromised due to a gain drop of the recording device. Light blue bars show blue whale acoustic presence [h d^-1^], black line shows mean daily sea-ice concentration [%], dark blue line shows mean daily SST [°C], yellow line shows mean daily ZMC [g/m^2^]. Sea-ice concentration, SST, and ZMC were averaged over a 60 km radius (eastern Fram Strait) and a 100 km radius (central Strait), based on maximum detection radii calculated by Ahonen et al. 2021 [19]. Dots indicate missing data for sea-ice concentration (black) and ZMC (yellow). Recorder ID mentioned in the subheadings above each subplot corresponds to recorders listed in Table 1. SST = sea surface temperature, ZMC = zooplankton mass content.

Blue whale acoustic presence showed a clear seasonality, with AB-calls being detected from late spring or summer (May to July) until autumn (October to November) (Fig 2). This period of seasonal acoustic presence often started with a few hours of presence per day, increasing in the following weeks. Most of acoustic presence hours (96%) were detected between July to October. Toward the end of the seasonal acoustic presence period, the number of acoustic presence hours decreased rather abruptly within a few days, with acoustic absence of blue whales from December throughout April in most recording years. However, in three years, blue whale vocalizations were detected in January (E2-2014, E4-2017), and February (E7-2021) on one or a few consecutive days. Blue whales were acoustically present in every recording year, except in 2015 when data collection commenced only in mid-October, resulting in a data gap for most of the year. No notable differences in temporal patterns of acoustic presence between eastern and central Fram Strait were observed.

**Fig 3:**
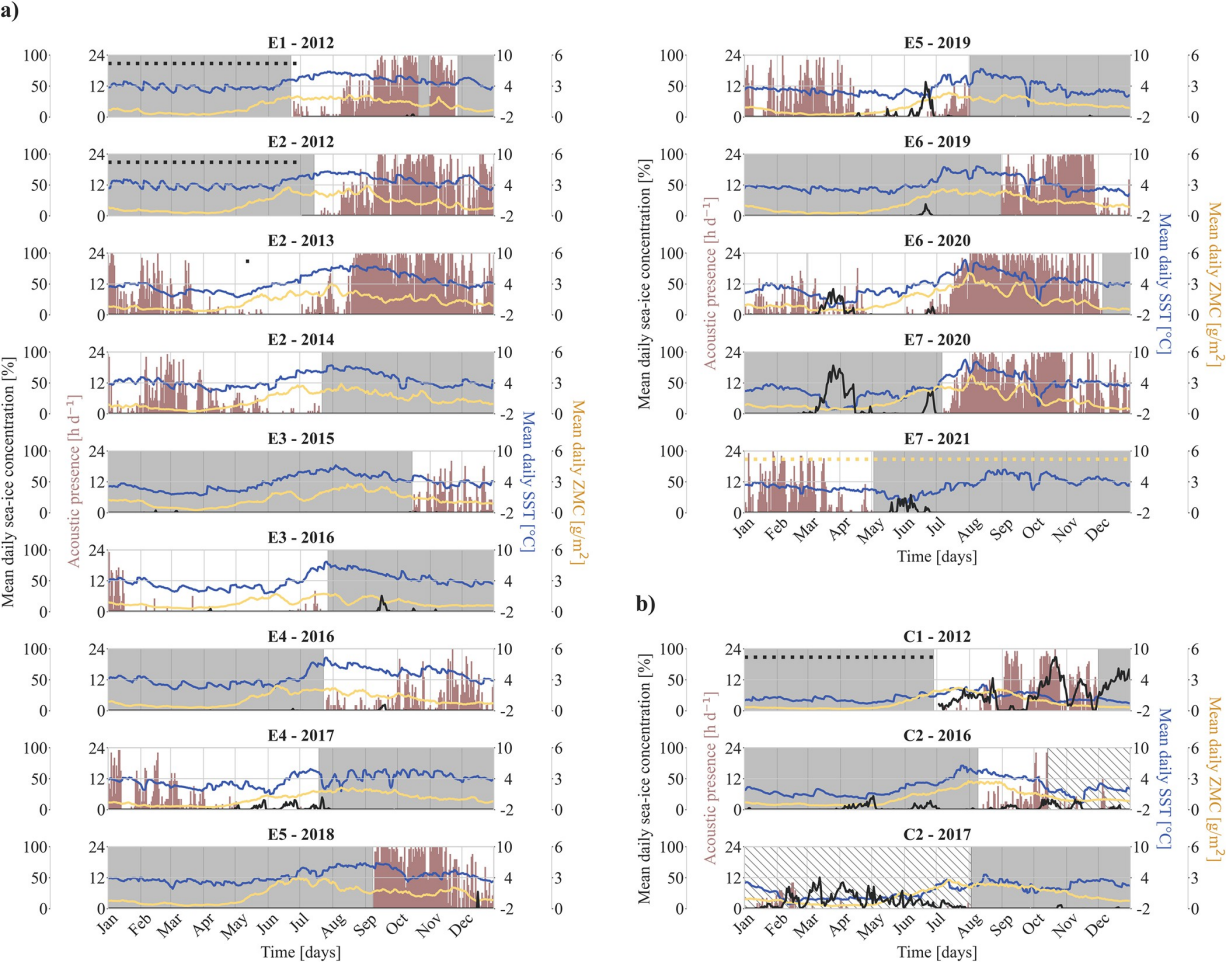
Interannual patterns in acoustic presence of fin whales (20 + 130 Hz pulses, 20 Hz pulses, and 130 Hz pulses) per recorder in a) eastern and b) central Fram Strait. Shaded periods denote missing acoustic data, while hatched areas indicate acoustic data that are presumably compromised due to a gain drop of the recording device. Red bars show fin whale acoustic presence [h d^-1^], black line shows mean daily sea-ice concentration [%], dark blue line shows mean daily SST [°C], yellow line shows mean daily ZMC [g/m^2^]. Sea-ice concentration, SST, and ZMC were averaged over a 30 km radius (eastern Fram Strait) and a 55 km radius (central Strait), based on maximum detection radii calculated by Ahonen et al. 2021 [19]. Dots indicate missing data for sea-ice concentration (black) and ZMC (yellow). Recorder ID mentioned in the subheadings above each subplot corresponds to recorders listed in Table 1. SST = sea surface temperature, ZMC = zooplankton mass content.

Fin whale acoustic presence (20 + 130 Hz pulses, 20 Hz pulses, and 130 Hz pulses) began to increase in summer (June to July), with pronounced acoustic presence in most recorders from July/August to November/December/January (Fig 3). Moderate acoustic presence was detected between January and April in all years. Between April and June, acoustic presence significantly decreased, with no call detections within one or two months in five years (E2-2013, E3-2016, E4-2017, E6-2020, C2-2017). Fin whale vocalizations were detected in all recording years. Temporal patterns of acoustic presence were similar between eastern and central Fram Strait, even though acoustic presence was clearly reduced in C2, where most data were presumably compromised due to a gain drop of the recording device.

**Fig 4:**
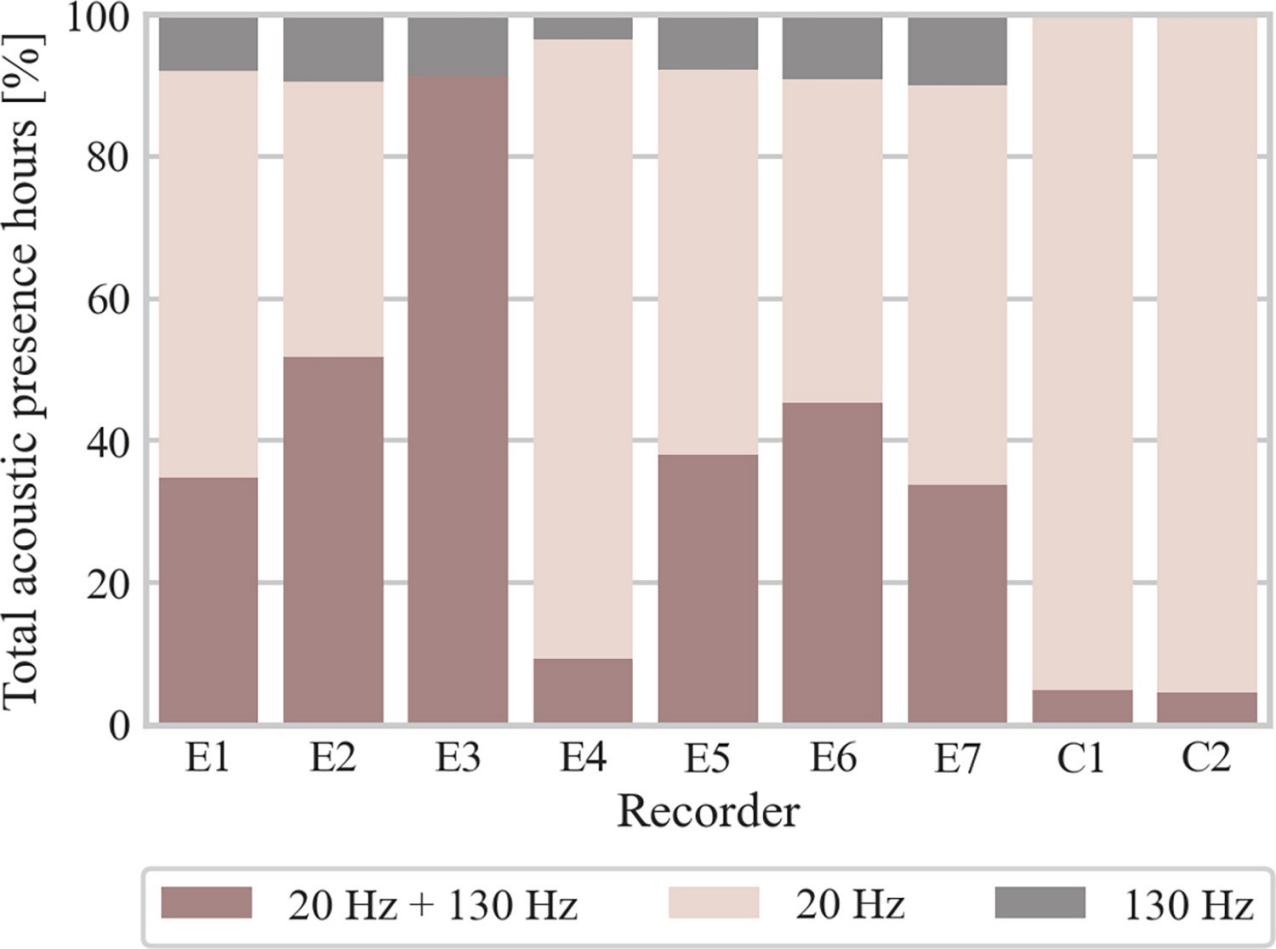
Percentage of acoustic presence hours with 20 and 130 Hz pulses simultaneously (red color, bottom), only 20 Hz pulses (beige color, middle), and only 130 Hz pulses (grey color, top). Anthropogenic noise, potentially masking 20 Hz pulses and resulting in exclusive detection of 130 Hz pulses during parts of the recording period, was present in all recorders.

Acoustic presence patterns for both 20 Hz and 130 Hz pulses followed similar trends, with the majority of acoustic presence hours occurring in late summer and autumn, along with occasional to frequent vocalizations during winter and spring (S3 Fig). Total fin whale acoustic presence comprised 52% of hours with only 20 Hz pulses, 40% of hours with both 20 and 130 Hz pulses simultaneously, and 8% of hours with only 130 Hz pulses. In most recorders, 130 Hz pulses (alone or in combination with 20 Hz pulses) were observed in > 42% of acoustic presence hours (Fig 4). However, in E4, C1, and C2, the occurrence was notably reduced, with 130 Hz pulses being detected in < 12% of acoustic presence hours. Anthropogenic noise in the lower frequencies (ship noise, seismic explorations) potentially masking 20 Hz pulses was discernable throughout all seasons, which most likely led to the 130 Hz-pulse-only detections.

### Environmental influence

**Fig 5:**
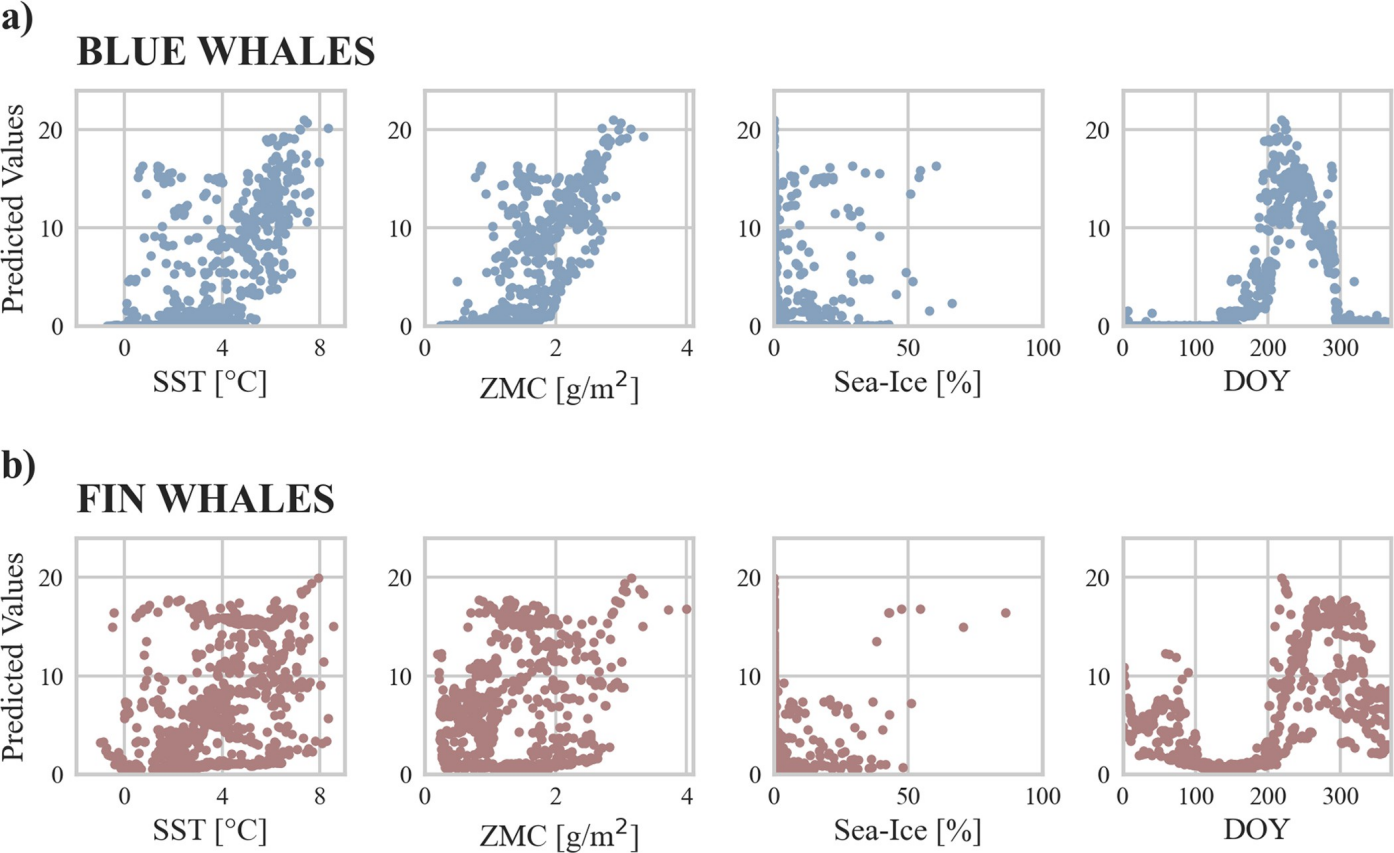
Random Forest partial effects of predictor variables on acoustic presence of blue whales (blue color) and fin whales (red color) in hours per day. SST = sea surface temperature, ZMC = zooplankton mass content, DOY = day of year.

The random forest model for blue whale acoustic presence achieved an R^2^ of 0.64 estimated on the validation dataset. SST, zooplankton mass content, and DOY were identified as the most influential predictors, each scoring 0.34 (SST), 0.30 (DOY), and 0.29 (zooplankton mass content) in feature importance (S4 Fig). Sensitivity testing showed minimal impact on model performance when replacing SST or zooplankton mass content with random values. However, replacement of DOY led to a drop in model performance to an R^2^ of 0.48. Partial effect analysis showed a positive correlation between acoustic presence and both zooplankton mass content and SST, as well as a distinct seasonal pattern linked to DOY (Fig 5).

Random forest model for fin whale acoustic presence had an R^2^ of 0.53 estimated on the validation dataset. DOY was identified as key predictor, scoring 0.62 in feature importance (S4 Fig). Replacement of DOY with random values led to a drop in model performance to an R^2^ of 0.23. For all other predictors, replacement with random values had reduced impact on model performance. Partial effects of DOY indicated a moderate increase of acoustic presence in winter and a strong peak in summer and autumn (Fig 5).

### Timing of onset and offset

**Fig 6:**
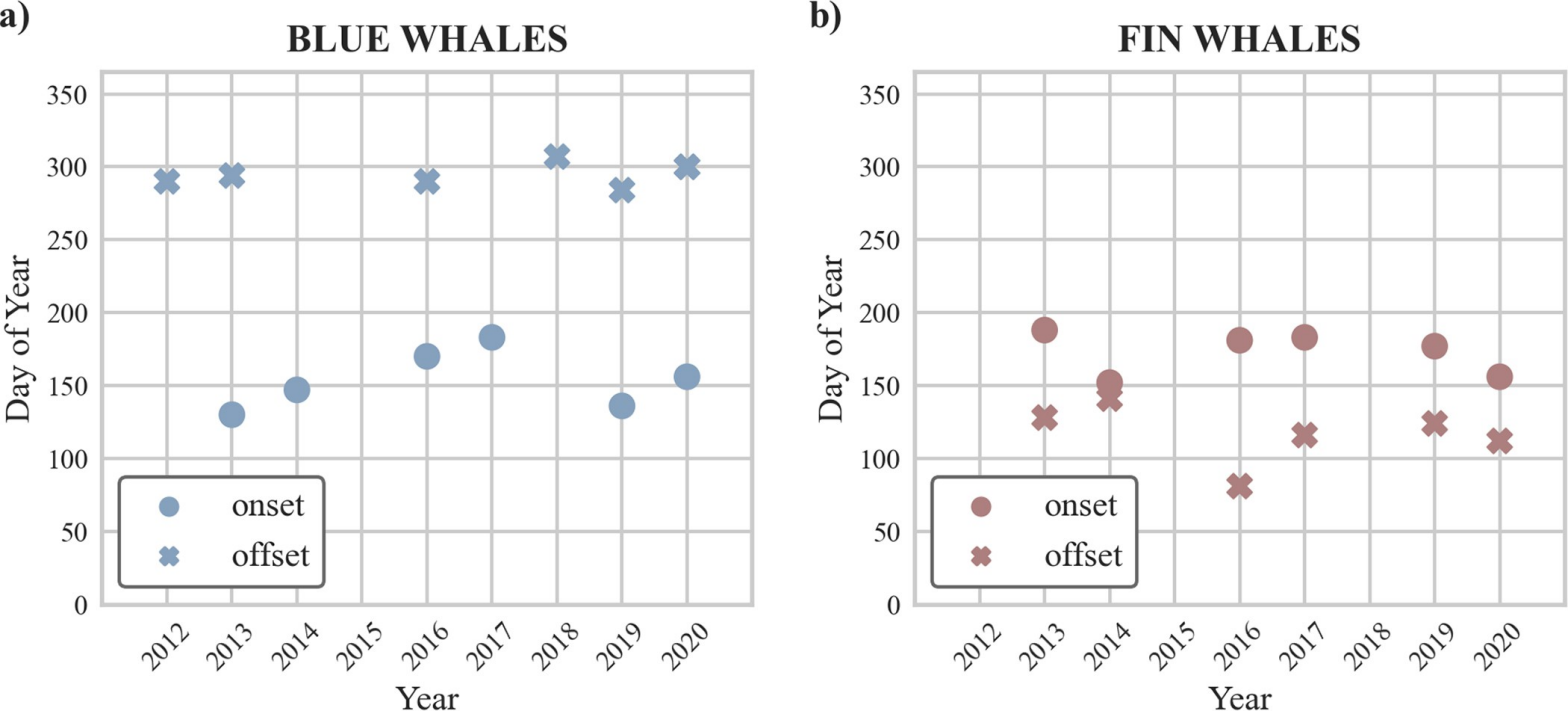
Onset and offset of seasonal acoustic presence periods of a) blue and b) fin whales per year. Ordinal date of onset and offset was determined by calculating the cumulative probability distribution of days with acoustic presence within a defined time period and extracting the 5^th^ (onset) and 95^th^ (offset) percentile. Circle indicates onset, cross indicates offset. Recorders and years included in the analysis were: blue whale onset - E2-2013, E2-2014, E3-2016, E4-2017, E5-2019, E6-2020; blue whale offset - E2-2012, E2-2013, E4-2016, E5-2018, E6-2019, E7-2020; fin whale onset - E2-2013, E2-2014, E3-2016, E4-2017, E5-2019, E6-2020; and fin whale offset - E2-2013, E2-2014, E3-2016, E4-2017, E5-2019, E6-2020.

The timing of the onset of blue whale acoustic presence in eastern Fram Strait varied between years, taking place between the 10^th^ of May (ordinal date: 130) and 2^nd^ of July (ordinal date: 183); while the timing of the offset was rather stable taking place between the 16^th^ of October (ordinal date: 290) and the 3^rd^ of November (ordinal date: 307) (Fig 6). For fin whales, both the timing of onset and offset in eastern Fram Strait showed interannual variations (Fig 6). The onsets took place between the 1^st^ of June (ordinal date: 152) and 7^th^ of July (ordinal date: 188); and offsets took place between the 21^st^ of March (ordinal date: 81) and 21^st^ of May (ordinal date: 141). No significant correlations were observed between the recording year and the ordinal date of onset/offset of the acoustic occurrence for both species.

## Discussion

Our investigation marks the first multi-year acoustic study of blue and fin whales in eastern Fram Strait. The area’s distinct conditions, shaped by the West Spitsbergen Current carrying warm Atlantic water northward and creating almost ice-free conditions year-round, offer a unique habitat for baleen whale species. Our findings provide crucial insights into acoustic presence patterns and environmental drivers, and establish a critical baseline for monitoring species occurrence in a vulnerable polar habitat already heavily affected by global warming.

### How do acoustic presence patterns vary across different Fram Strait sites?

Our study adds to previous acoustic research on blue and fin whales across Fram Strait, including two multi-year studies [19, 33] and several shorter ones [28, 32, 34, 42]. Below, we compare findings from different sites (west, central, east, Kongsfjorden; see S5 Fig for mooring positions). As blue and fin whales are recognized for vocalizing year-round [e.g. 37, 82, 83], acoustic presence is used as a proxy for physical presence, even though patterns of acoustic presence/absence might also reflect variations in calling behavior.

We observed a seasonal pattern in the occurrence of blue whale vocalizations in eastern Fram Strait, with increased acoustic presence between July and October (Fig 2). This indicates the animals primarily inhabit this region during summer and early autumn, between their arrival at and departure from the study area. Acoustic studies conducted in western and central Fram Strait between 2008 and 2018 reported a similar seasonal pattern, with most calls being detected from August to October [19, 32]. Pronounced acoustic presence occurred a month earlier in eastern Fram Strait (this study) compared to central and western Fram Strait [19, 32], suggesting that the eastern region is frequented by migratory individuals earlier than the central/western site. In Kongsfjorden, blue whale vocalizations were exclusively detected in late August 2017 by a study conducted between 2017 and 2018 [42], indicating rather restricted physical presence compared to other Fram Strait sites. However, when comparing blue whale acoustic presence across sites it should be considered that Ahonen et al. 2021 [19] provided call rates, Moore et al. 2012 [32] provided percentage of days/month with acoustic presence, and Llobet et al. 2023 [42] provided percentage of hours per week with acoustic presence; whereas our study used hours/day with acoustic presence. The use of different metrics by each study complicates direct comparisons. To better compare acoustic presences across sites and to study habitat use and migration patterns in (sub)Arctic waters, it would be beneficial to standardize the metrics used and establish collaborative research networks.

In eastern Fram Strait, we detected blue whale vocalizations during January and February in three years (2014, 2017, 2021) on one or more consecutive days (Fig 2). Vocalizations outside the seasonal acoustic presence period in summer and autumn were previously reported for this site, with calls being detected throughout December 2009 [34]. It indicates that individual blue whales prolong their seasonal stays, overwinter in, or swim into eastern Fram Strait during winter. In central Fram Strait, we did not detect any blue whale vocalizations between December and June, however, due to a hydrophone gain drop of C2, acoustic data are compromised from October 2016 onwards and blue whale presence might possibly be underestimated. However, in accordance with our findings, a previous study reported blue whale acoustic absence in central Fram Strait from November through May (between 2008 and 2012), and in western Fram Strait from January through March (between 2008 and 2018), suggesting the animals to not inhabit both sites at these times [19].

Our study showed increased acoustic presence of fin whales in eastern Fram Strait in summer and autumn, moderate acoustic presence during winter/early spring and strongly reduced acoustic presence or acoustic absence from mid-spring onwards (Fig 3). A similar pattern was previously reported in eastern Fram Strait between 2009 and 2010 by studies measuring the fin index, a relative measure for cumulative energy in the fin whale frequency band [28, 34]. Year-round fin whale acoustic presence was further reported for Kongsfjorden between 2014 and 2020 [33]. This indicates the eastern part of Fram Strait to be inhabited by fin whales during most of the year. In contrast, fin whale acoustic presence at western Fram Strait occurred sporadically throughout the year at low levels between 2012 and 2018 [19]. Differing acoustic presence between eastern and western Fram Strait suggest varying population densities, potentially indicating higher densities in the east. However, differences in acoustic presence might also reflect changes in calling behavior, or differences in detection ranges between sites.

Fin whale acoustic presence patterns reported in our study were similar in eastern and central Fram Strait, even though data for central Fram Strait was limited. Interestingly, we detected fin whale vocalizations in central Fram Strait between January and April 2017 (Fig 3), indicating individuals visiting the area during that time. A previous study conducted in central Fram Strait between 2008 and 2012 reported fin whale acoustic presence mostly from July through December, but also showed increased fin whale acoustic presence in January 2012 [19]. Extended acoustic monitoring in central Fram Strait is required to determine if acoustic presence during winter and spring is an anomaly, reflects normal interannual or spatial variation, or indicates a trend towards year-round acoustic presence.

### What drives acoustic presence of blue and fin whales in Fram Strait?

Differences in acoustic presence between eastern (this study) and western [19, 32] Fram Strait could be explained by varying environmental surroundings: While eastern Fram Strait is mainly influenced by the West Spitsbergen Current carrying warm Atlantic water northwards, western Fram Strait is heavily impacted by the East Greenland Current bringing cold water and ice masses southwards [30]. Temperature differences across Fram Strait lead to diverse ice conditions among sites. The milder and predominantly ice-free conditions in the eastern part of Fram Strait may provide favorable feeding and habitat conditions for seasonally migrating species throughout the year. This could explain differences in acoustic presence patterns compared to western and central Fram Strait, including earlier acoustic presence of blue whales, blue whale vocalizations during winter, as well as year-round and pronounced acoustic presence of fin whales in eastern Fram Strait [19, 28, 32, 34].

Random forest model for blue whale acoustic presence showed high impact of SST, zooplankton mass content, and DOY, with sensitivity testing suggesting SST and zooplankton mass content to be highly correlated (S4 Fig). As prey abundance is considered a primary factor governing baleen whale occurrences [e.g. 84–86], it is likely that zooplankton mass content directly influenced blue whale acoustic presence, while SST had indirect effects. Blue whales feed almost exclusively on euphausiids, mainly Arctic krill (*Thysanoessa raschii*) and northern krill (*Meganyctiphanes norvegica*) [26, 87]. One report from whalers suggested that blue whales follow high densities of zooplankton (mainly euphasiids) that are found along the edge of the continental shelf [88]. Another historic report described autumn departure of blue whales from the Arctic Ocean, including waters off Svalbard, to be tied to a decrease in food supply and not to a lowering of SST [26]. In our study, elevated zooplankton mass content levels observed during summer and autumn likely accounted for increased blue whale acoustic presence as indicated by the positive correlation between acoustic presence and modeled zooplankton in the random forest model. Sensitivity testing further revealed a strong influence of DOY, emphasizing a clear seasonality in blue whale acoustic presence.

In contrast to blue whales, fin whales are more flexible regarding their prey, generally preferring macro-zooplankton, such as euphausiids or copepods; but also preying on fish, such as capelin (*Mallotus villosus*) or herring (*Clupea harengus*) [27]. They are recognized for their ability to shift their distributions rapidly in response to changing prey fields [89, 90]. Likely due to their highly adaptable feeding habits, whaling reports describe spatio-temporal migration patterns of fin whales as more irregular compared to blue whales [25, 27]. The differences we observed in the acoustic presence of fin whales between years and locations are therefore probably linked to the animals responding to changes in prey availability. Our random forest models identified DOY as having the greatest impact on fin whale acoustic presence (S4 Fig). However, the overall model performance was lower than that of the blue whale model, indicating that the high feature importance score of DOY in the fin whale model should be interpreted with care. Environmental predictors we used for the random forest model showed little influence, probably because they did not sufficiently capture the effect of forage fish abundance. For example, occurrence of capelin along the west coast of Svalbard during winter [91] might attract fin whales and explain acoustic presence in eastern Fram Strait beyond the summer/autumn period. Changes in forage fish abundance could also lead to localized movements that do not align with large-scale north-south migrations, thereby impacting acoustic presence patterns in Fram Strait. Whaling reports noted fin whales to arrive off Finnmark in March and April when large shoals of spawning capelin were present [22, 25]. The animals were described to come from the north and northeast [22, 25], possibly departing, among other locations, from eastern Fram Strait. A localized movement like this could influence fin whale abundance in Fram Strait and explain the decrease of fin whale acoustic presence we observed during spring.

In sum, our findings suggest that eastern Fram Strait serves as a regular feeding ground for blue and fin whales, further emphasizing the significance of the region as a key habitat for marine mammal species [92]. Vocalizations recorded during winter from both blue and fin whales imply that part of the populations remain in or swim into eastern Fram Strait throughout the year, instead of participating in seasonal large-scale migrations. This provides additional evidence supporting the hypothesis of a complex and non-obligatory migration behavior among baleen whale species, likely influenced by factors such as partial or delayed migration based on age, sex, or reproductive stage [e.g. 37, 78, 93]. Similar migration behaviors have previously been described for multiple (sub-)species and populations, including Antarctic blue whales [37, 78] and North Atlantic fin whales [19, 33, 94]. To our knowledge, the sporadic occurrence of Atlantic blue whales in Arctic waters during winter months is previously undocumented. However, based on the results from our study it remains uncertain as to whether this behavior signals a new response to climate change.

### Do acoustic presence patterns point towards a trend of change in eastern Fram Strait?

Fram Strait already experiences ongoing Atlantification across diverse trophic levels [e.g. 21, 95–98]. As part of this process, baleen whale occurrence is likely to undergo significant changes, including temporal expansion of presence of seasonally migrating species in Arctic waters due to shifts in migration phenology [10]. Supporting this hypothesis, acoustic data from western Fram Strait suggest earlier migratory arrival of blue whales in recent years, with increased call rates observed in June and July from 2015 to 2018 compared to 2008 to 2014 [19].

We found no temporal trend of change when comparing onset/offset of seasonal acoustic presence periods over the years, but we identified the onset of blue and fin whales, as well as the offset of fin whales to be subject to interannual variations (Fig 6). Notably, we found a rather consistent timing of blue whale offset. We are confident that the reported acoustic presence has minimal errors, as automated detectors proved highly sensitive, and all data underwent manual post-processing to identify false positive detections. Similar methods to those we used in our study to determine onset/offset dates were previously successful in detecting delayed migratory departure of bowhead (*Balaena mysticetus*) and beluga whales (*Delphinapterus leucas*) from the Beaufort Sea [81, 99].

Potential temporal trends in acoustic presence might not be fully discernible in our study due to constraints in the study design. To compare the onset and offset of acoustic presences, we aggregated acoustic data collected at different mooring positions in eastern Fram Strait. We view this as a reasonable approach given the relatively short distances between mooring positions and the similar acoustic presence patterns observed between them, suggesting limited spatial effects. However, potential local effects at individual mooring positions should still be considered. We assumed sound propagation distances to be 60 km for blue whales and 30 km for fin whales [19]. Given that the distance between mooring positions in eastern Fram Strait ranged from approximately 20 to 70 km, detection radii of different recorders did not fully overlap. As a result, detected acoustic presence might have a local bias, potentially obscuring any temporal trends of change for the complete dataset. One example for this could be the comparatively late blue whale onset in 2016 and 2017. The respective acoustic data were collected by two recorders (E3, E4) deployed at relatively large water depths (> 2000 m) in eastern Fram Strait. It is likely that local productivity at these deep-water positions varies compared to positions directly at the continental shelf, which might explain the later occurrence of blue whale onset in both years.

Comparison with previous acoustic studies in eastern Fram Strait on blue and fin whales [28, 34] might help to uncover potential temporal trends of change. Our study showed blue whale onset between 2013 and 2020 to usually occur in May or June, with increased acoustic presence (> 12 hours per day) starting in July. In contrast, studies conducted earlier in 2009, also in eastern Fram Strait, showed acoustic presence commencing only in mid-July and reaching peak values between August and October [28, 34]. The earlier onset in recent years we observed in our study could indicate a trend of earlier blue whale arrivals in eastern Fram Strait. However, divergences between studies might also be attributed to regular interannual variation, local differences at recording positions or methodological differences. For fin whales, our study reported similar temporal acoustic presence patterns to those found in 2009 and 2010 in eastern Fram Strait [28, 34].

Spatial shifts in the occurrence of seasonally migrating species could not be investigated with the data available for our study but have previously been documented for Fram Strait [20, 21]. Sighting data from west of Svalbard suggests a recent shift in the distributions of blue, fin, humpback (*Megaptera novaeangliae*), and minke whales (*B*. *acutorostrata*) from the continental shelf break to fjords and coastal areas [20]. Furthermore, increased sightings of blue, fin, humpback, minke, and sei whales (*B*. *borealis*) in Svalbard waters lately indicated northward expansion of these species, even though this trend may be influenced by heightened observation efforts and growing population sizes [21]. To further investigate spatial range expansion of seasonally migrating species, multi-year acoustic studies along a north-south transect in Fram Strait are crucial.

## Conclusion

Our study highlights eastern and central Fram Strait as a vital habitat for blue and fin whales. We document blue whale acoustic occurrences from May to November, with sporadic detections during winter in eastern Fram Strait. Furthermore, we show fin whale acoustic presence for most months in both regions. The findings indicate Fram Strait to be an important feeding ground for blue and fin whales, and support a complex and non-obligatory migration behavior of both species. The ongoing Atlantification in the region is expected to impact baleen whale occurrences, potentially resulting in a temporal expansion of seasonal visits by migratory species. This study provides crucial insights into current occurrence patterns and aids in monitoring future distributional shifts related to climate change.

## Supporting information

S1 Table Passive acoustic recorders deployed and recovered in eastern and central Fram Strait by AWI. Recorder ID was assigned to recorders selected for the current study.
(DOCX)

S2 Table Input parameters and values used for the application of the Band Limited Energy Detector in Raven Pro 1.6.4 to detect Atlantic blue whale AB calls. Detailed explanations of terms are given in the Raven software manual provided by Cornell Lab of Ornithology [64].
(DOCX)

S3 Table Assessment of automatic detector performance for detecting Atlantic blue whale AB calls on hourly basis in the test dataset, separated by recorder. After running the detector on the complete dataset, all acoustic files containing detections underwent manual verification for false positives, resulting in a final count of zero false positive hours for all recorders.TP = True Positives, FP = False Positives, TN = True Negatives, FN = False Negatives.
(DOCX)

S4 Table Threshold values tested per metric to asses optimal threshold combinations. SNR = signal to noise ratio.
(DOCX)

S5 Table Optimal threshold combination (true positive rate on a call basis greater than 0.7 while maintaining the minimum false positive rate) per recorder. Kurt = Kurtosis, KurtProd = Kurtosis Product, SNRT = temporal signal to noise ratio, SNRF = spectral signal to noise ratio, BW = bandwidth. NOAA PMEL data was not included in the analysis as it became available at a later stage.
(DOCX)

S6 Table Threshold combination applied to automatically detect fin whale 20 Hz pulses using the algorithm provided by Schall and Parcerisas [52]. Kurt = Kurtosis, KurtProd = Kurtosis Product, SNRT = temporal signal-to-noise ratio, SNRF = spectral signal-to-noise ratio, BW = bandwidth. Given the strong variations in optimal SNRF thresholds among different recorders, they were organized into two groups. Recorder group 1 comprises E1, E2, E5, E6, E7, and C1; while group 2 comprises E3, E4, and C2.
(DOCX)

S7 Table Assessment of automatic detector performance for detecting fin whale 20 Hz pulses on hourly basis in the test dataset, separated by recorder. After running the detector on the complete dataset, all acoustic files containing detections underwent manual verification for false positives, resulting in a final count of zero false positive hours for all recorders. TP = True Positives, FP = False Positives, TN = True Negatives, FN = False Negatives.
(DOCX)

S8 Table Parameter grid for hyperparameter tuning. A grid search with 5-fold cross-validation was executed to identify optimal parameter combinations maximizing model performances. Grid search was conducted in Python 3.11 using GridSearchCV from sklearn.model_selection. To decrease the risk of overfitting, max_depth was limited at 5.
(DOCX)

S1 Fig Distribution of metrics calculated by the automatic detector for fin whale 20 Hz pulses [65] for true fin whale 20 Hz pulses manually logged within the test dataset. To enhance the figure’s readability, the upper range of the Kurtosis Product was truncated at 600, even though the true upper limit stands at 4068.
(TIF)

S2 Fig Cumulative distribution of days with blue whale (top row) and fin whale (bottom row) vocalizations within specified time periods for each year and recorder. Analyzed days included May 1^st^ to July 31^st^ for blue whale onset; October 1^st^ to November 30^th^ for blue whale offset; 1^st^ of June to 31^st^ of July for fin whale onset; and 1^st^ of January to 31^st^ of May for fin whale offset. Onset was defined as the 5% quantile and offset as the 95% quantile of the cumulative distribution of days with acoustic presence in each time period.
(TIF)

S3 Fig Interannual patterns in acoustic presence of fin whales in a) eastern and b) central Fram Strait. Red bars indicate simultaneous presence of 20 Hz and 130 Hz calls, beige bars refer to exclusive presence of 20 Hz calls and grey bars indicate the exclusive presence of 130 Hz calls. Shaded regions denote missing data, while parallel lines indicate compromised acoustic data due to hydrophone gain drop. Recorder ID mentioned in the subheadings above each subplot corresponds to recorders listed in Table 1.
(TIF)

S4 Fig Relative importance of features for random forest models predicting a) blue and b) fin whale acoustic presence. SST = sea surface temperature, DOY = Day of the Year, ZMC = zooplankton mass content, Sea-Ice = sea-ice concentration.
(TIF)

S5 Fig Recorder positions in western, central, and eastern Fram Strait, as well as in Kongsfjorden, utilized for the current and for previous studies on the acoustic presence of blue and fin whales. The respective study is indicated by color. Bathymetry map was created in PyGMT v0.9.0 [46] using the SRTM15+V2.6 grid [47].
(TIF)
