# Supplementary material for: A multi-year analysis of acoustic occurrence and habitat use of blue and fin whales in eastern and central Fram Strait
Source: PLoS One. 2024 Nov 26;19(11):e0314369. doi: 10.1371/journal.pone.0314369 (PMC11594435; doi:10.1371/journal.pone.0314369)
Supplement: S1 Table — Recorder ID was assigned to recorders selected for the current study. (DOCX) [file pone.0314369.s001.docx]

| **Mooring ID** | **Recorder Serial Number** | **Recorder ID** | **Latitude [°]** | **Longitude [°]** | **Start Recording** | **End Recording** |
| --- | --- | --- | --- | --- | --- | --- |
| ARKF03-16 | SV1095 | - | 78,8308 | 8,0133 | 2015-10-02 | 2016-07-24 |
| ARKF04-15 | SV1024 | - | 78,8335 | 6,9998 | 2012-06-22 | 2012-12-11 |
| ARKF04-15 | SV1026 | E1 | 78,8335 | 6,9998 | 2012-06-22 | 2012-11-26 |
| ARKF04-19 | SV1088 | E6 | 78,9997 | 6,9997 | 2019-09-01 | 2020-12-04 |
| ARKF04-OZA | SV1096 | E5 | 79,1665 | 6,3327 | 2018-09-08 | 2019-08-01 |
| ARKF04-OZA2 | AU0302 | E7 | 79,1669 | 6,3327 | 2020-07-07 | 2021-05-02 |
| ARKF05-16 | SV1096 | - | 78,8333 | 6,0097 | 2015-10-04 | 2016-07-25 |
| ARKF05-17 | SV1088 | E4 | 79,0002 | 5,6687 | 2016-07-23 | 2017-07-18 |
| ARKF05-17 | SV1025 | - | 79,0002 | 5,6687 | 2016-07-23 | 2017-07-19 |
| ARKF05-17 | SV1054 | - | 79,0002 | 5,6687 | 2016-07-23 | 2017-04-12 |
| ARKF06-17 | SV1101 | - | 78,8453 | 4,6410 | 2015-10-04 | 2016-07-25 |
| ARKF16-09 | SV1021 | C1 | 78,8293 | 0,4295 | 2012-06-28 | 2012-11-30 |
| ARKF16-09 | SV1022 | - | 78,8293 | 0,4295 | 2012-06-28 | 2012-11-23 |
| ARKF16-09 | SV1023 | - | 78,8293 | 0,4295 | 2012-06-28 | 2012-12-07 |
| ARKR01-01 | SV1097 | C2 | 78,1702 | 0,0007 | 2016-08-09 | 2017-08-02 |
| ARKR02-01 | SV1091 | - | 78,8335 | 0,0015 | 2016-07-27 | 2017-07-21 |
