# Supplementary material for: A multi-year analysis of acoustic occurrence and habitat use of blue and fin whales in eastern and central Fram Strait
Source: PLoS One. 2024 Nov 26;19(11):e0314369. doi: 10.1371/journal.pone.0314369 (PMC11594435; doi:10.1371/journal.pone.0314369)
Supplement: S2 Table — Detailed explanations of terms are given in the Raven software manual provided by Cornell Lab of Ornithology [64]. (DOCX) [file pone.0314369.s002.docx]

| **Input Parameter** | **Value** |
| --- | --- |
| Minimum Frequency [Hz] | 16 |
| Maximum Frequency [Hz] | 18 |
| Minimum Duration [s] | 5.2 |
| Maximum Duration [s] | 20.15 |
| Minimum Separation [s] | 0.65 |
| Minimum Occupancy [%] | 70 |
| Return Occupancy | yes |
| Signal-to-Noise Threshold [dB] | 4 (above) |
| Noise Block Size [s] | 79.95 |
| Noise Hop Size [s] | 40.3 |
| Noise Percentile | 20 |
