# Supplementary material for: A multi-year analysis of acoustic occurrence and habitat use of blue and fin whales in eastern and central Fram Strait
Source: PLoS One. 2024 Nov 26;19(11):e0314369. doi: 10.1371/journal.pone.0314369 (PMC11594435; doi:10.1371/journal.pone.0314369)
Supplement: S3 Table — After running the detector on the complete dataset, all acoustic files containing detections underwent manual verification for false positives, resulting in a final count of zero false positive hours for all recorders.TP = True Positives, FP = False Positives, TN = True Negatives, FN = False Negatives. (DOCX) [file pone.0314369.s003.docx]

| **Recorder ID** | **# TP** | **# FP** | **# TN** | **# FN** | **Sensitivity** | **Precision** |
| --- | --- | --- | --- | --- | --- | --- |
| E1 | 86 | 14 | 178 | 0 | 1 | 0.86 |
| E2 | 104 | 18 | 1061 | 5 | 0.95 | 0.85 |
| E3 | 7 | 6 | 465 | 2 | 0.78 | 0.54 |
| E4 | 88 | 42 | 489 | 0 | 1 | 0.68 |
| E5 | 15 | 28 | 507 | 0 | 1 | 0.35 |
| E6 | 132 | 25 | 577 | 0 | 1 | 0.84 |
| E7 | 49 | 7 | 305 | 3 | 0.94 | 0.88 |
| C1 | 85 | 34 | 161 | 2 | 0.98 | 0.71 |
| C2 | 30 | 37 | 537 | 1 | 0.97 | 0.45 |
