# Supplementary material for: A multi-year analysis of acoustic occurrence and habitat use of blue and fin whales in eastern and central Fram Strait
Source: PLoS One. 2024 Nov 26;19(11):e0314369. doi: 10.1371/journal.pone.0314369 (PMC11594435; doi:10.1371/journal.pone.0314369)
Supplement: S4 Table — SNR = signal to noise ratio. (DOCX) [file pone.0314369.s004.docx]

| **Parameter** | **Threshold Values** |
| --- | --- |
| Kurtosis 1 | 2.5, 3.0, 3.5 |
| Kurtosis 2 | 4.0, 4.5, 5.0 |
| Kurtosis-Product | 20, 25, 30 |
| Temporal SNR 1 | -6, -4, -2, 0, 2, 4 |
| Temporal SNR 2 | -16, -14, -12, -10, -8 |
| Spectral SNR | -7, -5, -3, -1, 1, 3, 5, 7, 9 |
| Bandwidth | 10, 14, 18 |
