# Supplementary material for: A multi-year analysis of acoustic occurrence and habitat use of blue and fin whales in eastern and central Fram Strait
Source: PLoS One. 2024 Nov 26;19(11):e0314369. doi: 10.1371/journal.pone.0314369 (PMC11594435; doi:10.1371/journal.pone.0314369)
Supplement: S5 Table — Kurt = Kurtosis, KurtProd = Kurtosis Product, SNRT = temporal signal to noise ratio, SNRF = spectral signal to noise ratio, BW = bandwidth. NOAA PMEL data was not included in the analysis as it became available at a later stage. (DOCX) [file pone.0314369.s005.docx]

|  | **Kurt1** | **Kurt2** | **KurtProd** | **SNRT1** | **SNRT2** | **SNRF** | **BW** |
| --- | --- | --- | --- | --- | --- | --- | --- |
| **E1** | 3.5 | 4.5 | 30 | -2 | -16 | 9 | 10 |
| **E4** | 3 | 4 | 30 | -6 | -10 | -7 | 10 |
| **E5** | 3.5 | 4 | 25 | -6 | -14 | 7 | 10 |
| **E6** | 3 | 4 | 30 | -6 | -16 | 7 | 10 |
| **E7** | 3.5 | 4 | 20 | -6 | -16 | 5 | 10 |
| **C1** | 3.5 | 4 | 20 | -6 | -14 | 9 | 10 |
| **C2** | 3.5 | 4 | 25 | -6 | -12 | -3 | 18 |
