# Supplementary material for: A multi-year analysis of acoustic occurrence and habitat use of blue and fin whales in eastern and central Fram Strait
Source: PLoS One. 2024 Nov 26;19(11):e0314369. doi: 10.1371/journal.pone.0314369 (PMC11594435; doi:10.1371/journal.pone.0314369)
Supplement: S6 Table — Kurt = Kurtosis, KurtProd = Kurtosis Product, SNRT = temporal signal-to-noise ratio, SNRF = spectral signal-to-noise ratio, BW = bandwidth. Given the strong variations in optimal SNRF thresholds among different recorders, they were organized into two groups. Recorder group 1 comprises E1, E2, E5, E6, E7, and C1; while group 2 comprises E3, E4, and C2. (DOCX) [file pone.0314369.s006.docx]

|  | **Kurt1** | **Kurt2** | **KurtProd** | **SNRT1** | **SNRT2** | **SNRF** | **BW** |
| --- | --- | --- | --- | --- | --- | --- | --- |
| **Group 1** | 3.5 | 4 | 25 | -6 | -12 | 7 | 10 |
| **Group 2** | 2.5 | 4.5 | 20 | -6 | -14 | -7 | 10 |
