# Supplementary material for: A multi-year analysis of acoustic occurrence and habitat use of blue and fin whales in eastern and central Fram Strait
Source: PLoS One. 2024 Nov 26;19(11):e0314369. doi: 10.1371/journal.pone.0314369 (PMC11594435; doi:10.1371/journal.pone.0314369)
Supplement: S7 Table — After running the detector on the complete dataset, all acoustic files containing detections underwent manual verification for false positives, resulting in a final count of zero false positive hours for all recorders. TP = True Positives, FP = False Positives, TN = True Negatives, FN = False Negatives. (DOCX) [file pone.0314369.s007.docx]

|  | **# TP** | **# FP** | **# TN** | **# FN** | **Sensitivity** | **Precision** | **Group** |
| --- | --- | --- | --- | --- | --- | --- | --- |
| E1 | 78 | 103 | 94 | 3 | 0.96 | 0.43 | 1 |
| E2 | 347 | 216 | 568 | 57 | 0.86 | 0.62 | 1 |
| E3 | 39 | 393 | 48 | 0 | 1 | 0.09 | 2 |
| E4 | 103 | 504 | 10 | 2 | 0.98 | 0.17 | 2 |
| E5 | 169 | 140 | 200 | 41 | 0.8 | 0.54 | 1 |
| E6 | 279 | 186 | 234 | 35 | 0.89 | 0.6 | 1 |
| E7 | 104 | 44 | 195 | 21 | 0.83 | 0.7 | 1 |
| C1 | 76 | 85 | 108 | 13 | 0.85 | 0.47 | 1 |
| C2 | 14 | 588 | 3 | 0 | 1 | 0.02 | 2 |
