# Supplementary material for: A multi-year analysis of acoustic occurrence and habitat use of blue and fin whales in eastern and central Fram Strait
Source: PLoS One. 2024 Nov 26;19(11):e0314369. doi: 10.1371/journal.pone.0314369 (PMC11594435; doi:10.1371/journal.pone.0314369)
Supplement: S8 Table — A grid search with 5-fold cross-validation was executed to identify optimal parameter combinations maximizing model performances. Grid search was conducted in Python 3.11 using GridSearchCV from sklearn.model_selection. To decrease the risk of overfitting, max_depth was limited at 5. (DOCX) [file pone.0314369.s008.docx]

| **Hyperparameter** | **Defined range** |
| --- | --- |
| 'n_estimators' | 200, 300, 400, 500 |
| 'max_depth' | 1, 2, 3, 4, 5 |
| 'min_samples_split' | 2, 3, 4, 5, 6, 7, 8, 9, 10 |
| 'min_samples_leaf' | 1, 2, 3, 4, 5, 6 |
| 'max_features' | 1, 2, 3 |
