# Supplementary figures and images for: A multi-year analysis of acoustic occurrence and habitat use of blue and fin whales in eastern and central Fram Strait
Source: PLoS One. 2024 Nov 26;19(11):e0314369. doi: 10.1371/journal.pone.0314369 (PMC11594435; doi:10.1371/journal.pone.0314369)

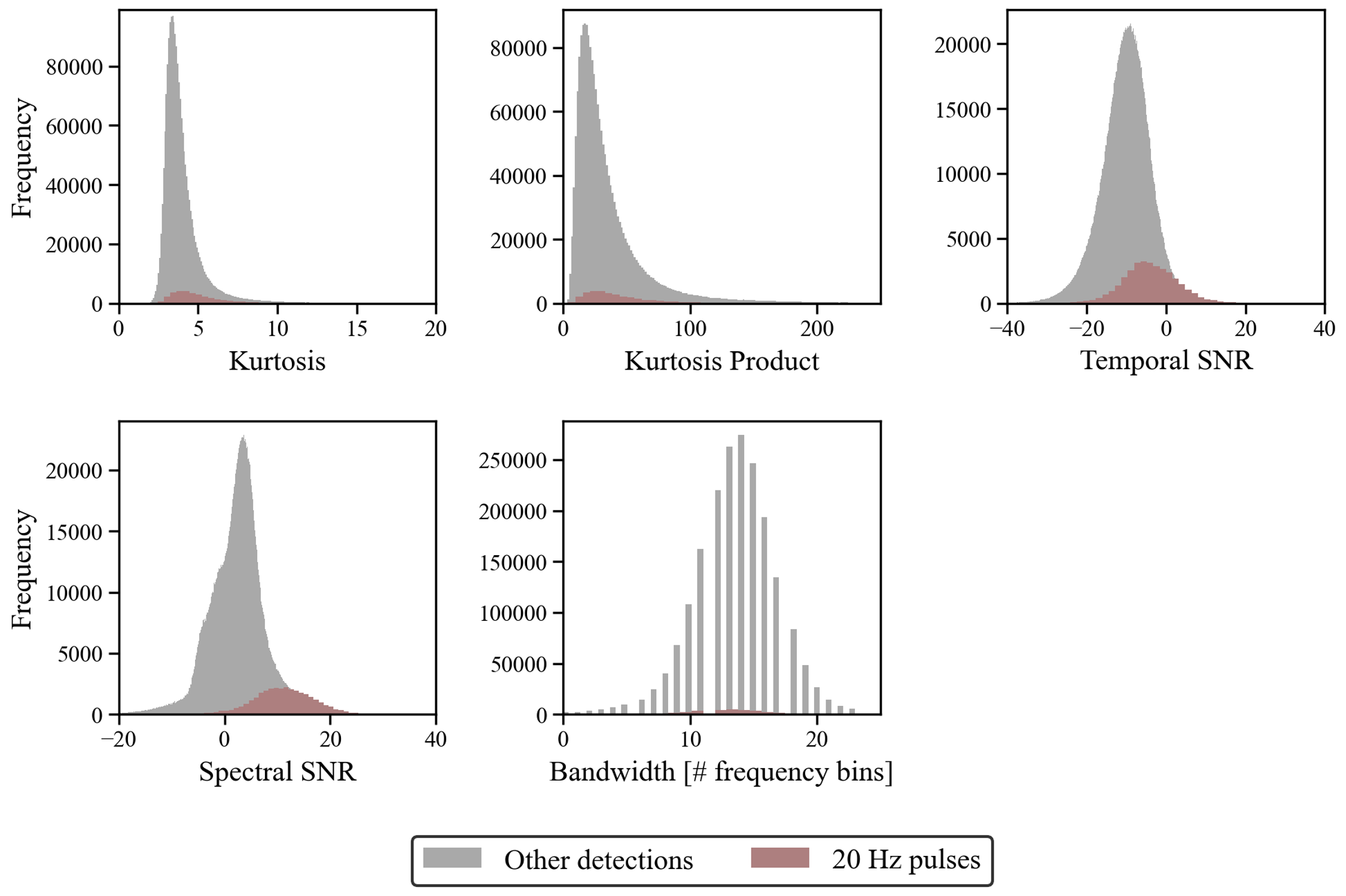

Supplement: S1 Fig — To enhance the figure’s readability, the upper range of the Kurtosis Product was truncated at 600, even though the true upper limit stands at 4068. (TIF) [file pone.0314369.s009.tif]

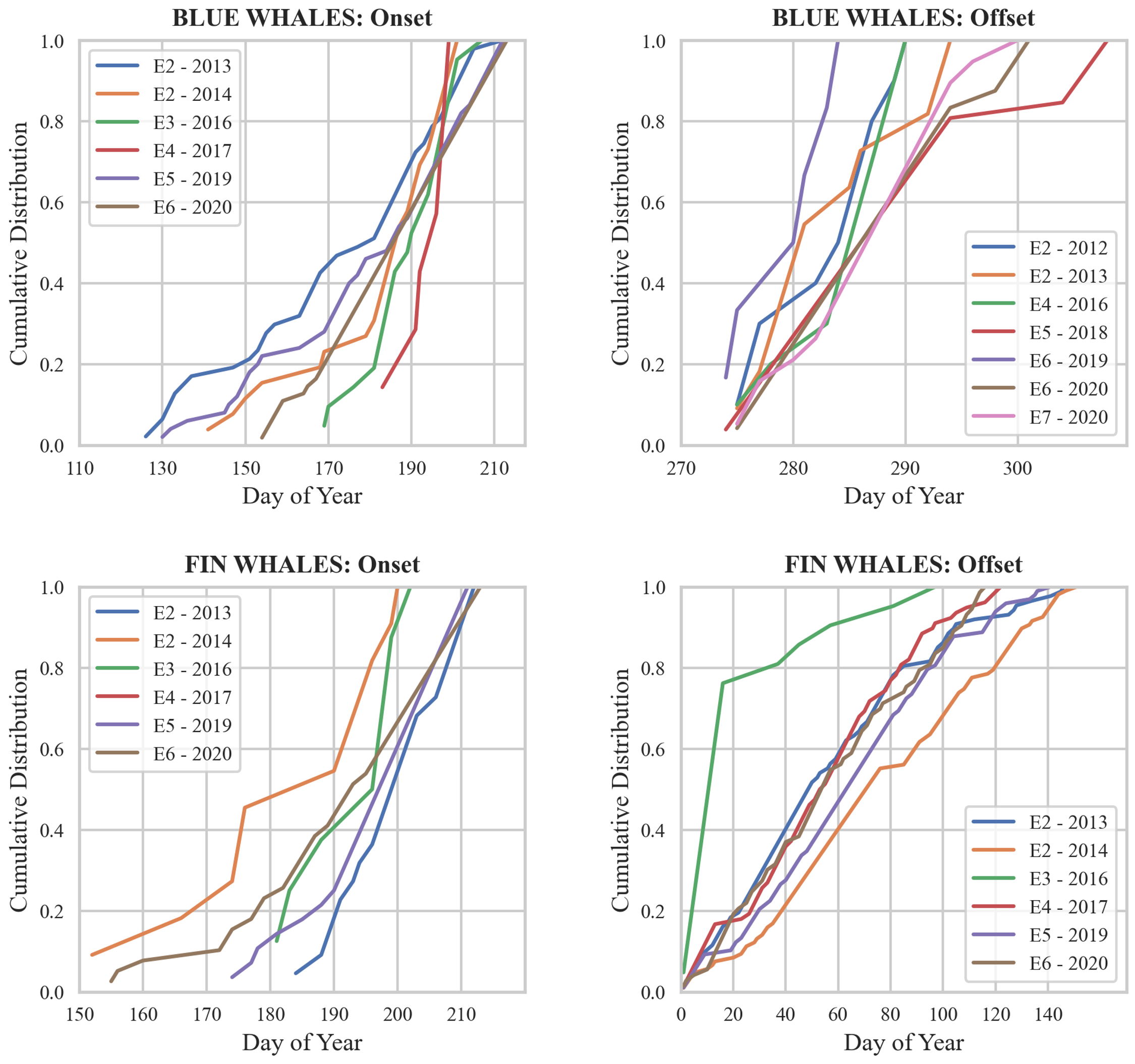

Supplement: S2 Fig — Cumulative distribution of days with blue whale (top row) and fin whale (bottom row) vocalizations within specified time periods for each year and recorder. Analyzed days included May 1st to July 31st for blue whale onset; October 1st to November 30th for blue whale offset; 1st of June to 31st of July for fin whale onset; and 1st of January to 31st of May for fin whale offset. Onset was defined as the 5% quantile and offset as the 95% quantile of the cumulative distribution of days with acoustic presence in each time period. (TIF) [file pone.0314369.s010.tif]

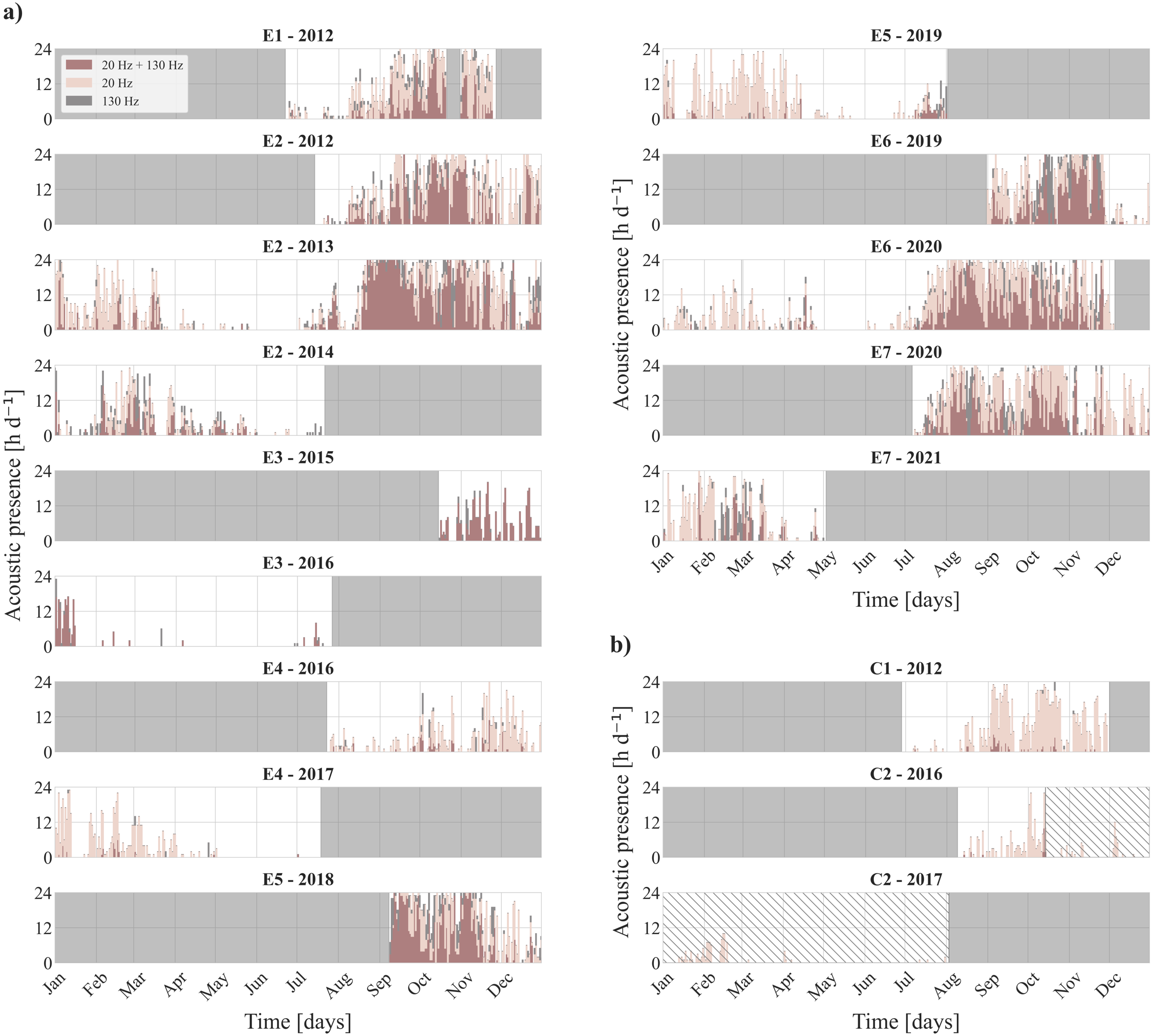

Supplement: S3 Fig — Interannual patterns in acoustic presence of fin whales in a) eastern and b) central Fram Strait. Red bars indicate simultaneous presence of 20 Hz and 130 Hz calls, beige bars refer to exclusive presence of 20 Hz calls and grey bars indicate the exclusive presence of 130 Hz calls. Shaded regions denote missing data, while parallel lines indicate compromised acoustic data due to hydrophone gain drop. Recorder ID mentioned in the subheadings above each subplot corresponds to recorders listed in Table 1. (TIF) [file pone.0314369.s011.tif]

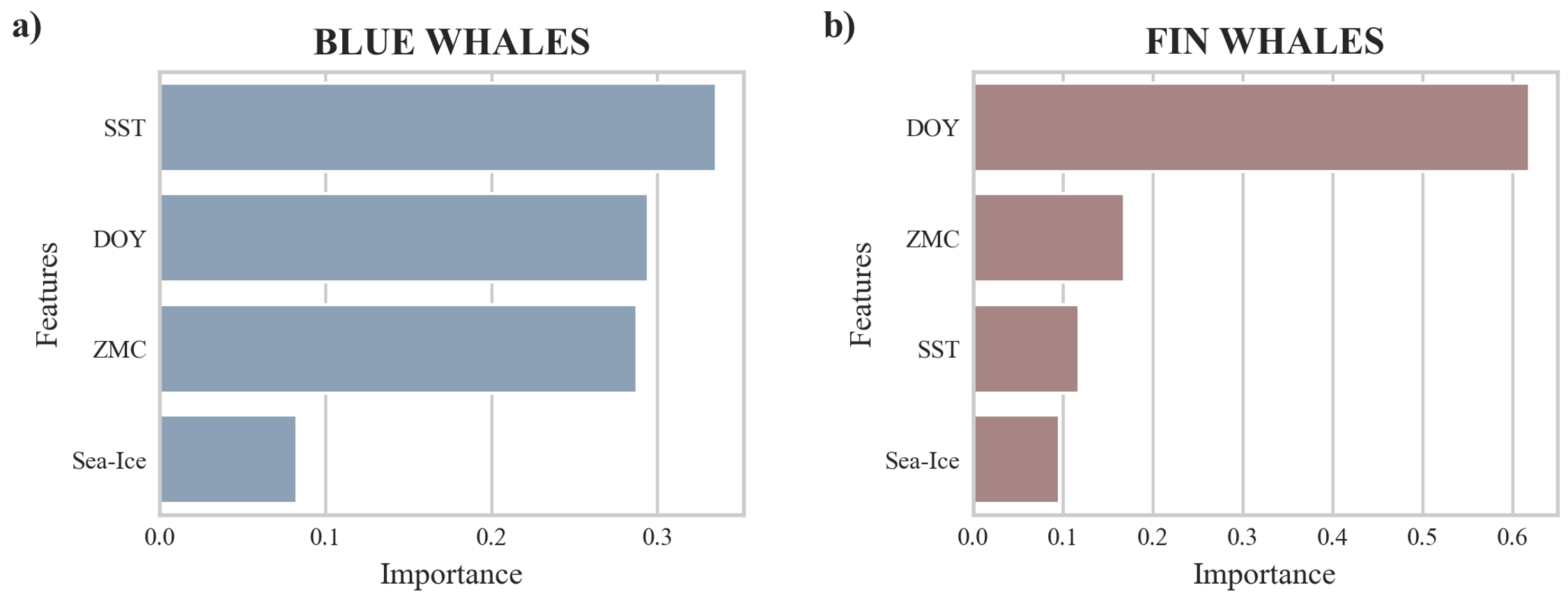

Supplement: S4 Fig — Relative importance of features for random forest models predicting a) blue and b) fin whale acoustic presence. SST = sea surface temperature, DOY = Day of the Year, ZMC = zooplankton mass content, Sea-Ice = sea-ice concentration. (TIF) [file pone.0314369.s012.tif]

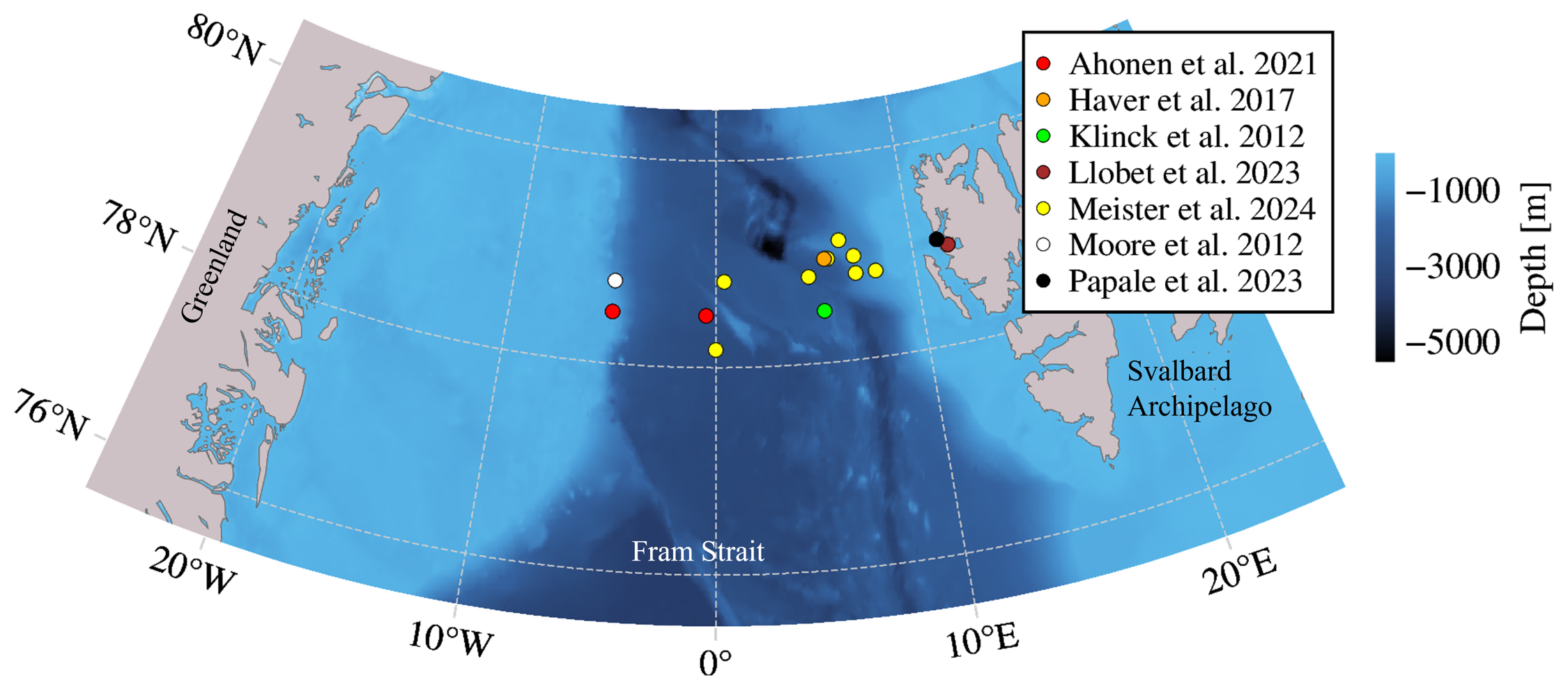

Supplement: S5 Fig — The respective study is indicated by color. Bathymetry map was created in PyGMT v0.9.0 [46] using the SRTM15+V2.6 grid [47]. (TIF) [file pone.0314369.s013.tif]
